# Hypoxia Promotes Uveal Melanoma Invasion through Enhanced Notch and MAPK Activation

**DOI:** 10.1371/journal.pone.0105372

**Published:** 2014-08-28

**Authors:** Laura Asnaghi, Michael H. Lin, Kah Suan Lim, Kah Jing Lim, Arushi Tripathy, Murilo Wendeborn, Shannath L. Merbs, James T. Handa, Akrit Sodhi, Eli E. Bar, Charles G. Eberhart

**Affiliations:** 1 Department of Pathology, Johns Hopkins University, School of Medicine, Baltimore, Maryland, United States of America; 2 Department of Ophthalmology, Johns Hopkins University, School of Medicine, Baltimore, Maryland, United States of America; 3 Department of Oncology, Johns Hopkins University, School of Medicine, Baltimore, Maryland, United States of America; 4 Department of Neurological Surgery, School of Medicine, Case Western Reserve University, Cleveland, Ohio, United States of America; University of California at Davis, United States of America

## Abstract

The transcriptional response promoted by hypoxia-inducible factors has been associated with metastatic spread of uveal melanoma. We found expression of hypoxia-inducible factor 1α (HIF-1α) protein in well-vascularized tumor regions as well as in four cell lines grown in normoxia, thus this pathway may be important even in well-oxygenated uveal melanoma cells. HIF-1α protein accumulation in normoxia was inhibited by rapamycin. As expected, hypoxia (1% pO_2_) further induced HIF-1α protein levels along with its target genes VEGF and LOX. Growth in hypoxia significantly increased cellular invasion of all 5 uveal melanoma lines tested, as did the introduction of an oxygen-insensitive HIF-1α mutant into Mel285 cells with low HIF-1α baseline levels. In contrast, HIF-1α knockdown using shRNA significantly decreased growth in hypoxia, and reduced by more than 50% tumor invasion in four lines with high HIF-1α baseline levels. Pharmacologic blockade of HIF-1α protein expression using digoxin dramatically suppressed cellular invasion both in normoxia and in hypoxia. We found that Notch pathway components, including Jag1-2 ligands, Hes1-Hey1 targets and the intracellular domain of Notch1, were increased in hypoxia, as well as the phosphorylation levels of Erk1-2 and Akt. Pharmacologic and genetic inhibition of Notch largely blocked the hypoxic induction of invasion as did the pharmacologic suppression of Erk1-2 activity. In addition, the increase in Erk1-2 and Akt phosphorylation by hypoxia was partially reduced by inhibiting Notch signaling. Our findings support the functional importance of HIF-1α signaling in promoting the invasive capacity of uveal melanoma cells in both hypoxia and normoxia, and suggest that pharmacologically targeting HIF-1α pathway directly or through blockade of Notch or Erk1-2 pathways can slow tumor spread.

## Introduction

Melanomas arising in the uveal tract of the eye represent the most common primary intraocular cancer in adults, and half spread hematogenously to visceral organs, generally leading to patient death. Approximately 1500 new uveal melanoma cases are diagnosed in the United States each year, accounting for 5% of all melanomas and 13% of melanoma deaths [1]. Treatment depends upon the size of the tumor and includes enucleation, brachytherapy, transpupillary thermotherapy, and local resection [2], [3]. Despite these treatments, metastasis remains a critical problem, and improved understanding of the signaling pathways driving tumor dissemination and facilitating growth at distant sites is needed.

The most significant single chromosomal marker of poor outcome in uveal melanoma is loss of one copy of chromosome 3 [4]–[8], while activating mutations in the alpha subunit of heterotrimeric G proteins, GNAQ or GNA11, are considered an early event in the development of the disease [9]. Recently, inactivating mutations in the tumor suppressor BRCA1-associated protein-1 (*BAP1*), located at 3p21.1, were shown to occur almost exclusively in metastatic uveal melanomas with monosomy 3 [10]. Finally, expression analysis has revealed two separate groups of uveal melanoma: class 1 tumors with low metastatic risk, and class 2 tumors, characterized by metastatic spread and worse prognosis [11], [12].

Interestingly, one biomarker strongly associated with class 2 signature is the Hypoxia-Inducible Factor 1α (HIF-1α), the main regulator of the hypoxic response [13]. Hypoxia leads to the transcriptional induction of genes that participate in angiogenesis, iron metabolism, glucose metabolism and cell proliferation/survival. The main factor mediating these responses is HIF-1α, an oxygen-sensitive transcriptional activator regulated by oxygen-dependent post-translational modifications, including hydroxylation in Pro-402 and Pro-564, catalyzed by HIF-1α prolyl-hydroxylases (PHD1,2,3) [14]. Under hypoxic conditions, proline hydroxylation is suppressed, stabilizing the HIF-1α subunit, which translocates to the nucleus, where it binds HIF-1β, forming a complex, that activates gene transcription by interacting with hypoxic response elements (HRE) in the regulatory region of the target genes [15]. HIF-1α contributes to cancer progression by activating a transcriptional program, which includes glycolytic enzymes, proangiogenic proteins and motility factors, enabling tumors to grow, invade and metastasize in the hostile environment characterized by low oxygen tension [16]. Another HIF-α subunit, HIF-2α, can also play an important role in the hypoxic response [17]. While HIF-1α is ubiquitously expressed, HIF-2α is confined to the endothelium, kidney, heart, lung and small intestine, activating preferentially the expression of lysyl-oxidase, transforming growth factor-α (TGF-α) and Cyclin D1, and enhancing the transcriptional activity of c-myc [18], [19].

HIF-1α protein is highly expressed in metastatic uveal melanomas [13]. VEGF, a transcriptional target of HIF-1α, has also been associated with aggressive behaviour in uveal melanoma [20]. In addition, expression of another HIF-1α target gene, lysyl oxidase (LOX), was found to be elevated in aggressive epithelioid cell type and was associated with shorter metastasis-free survival, suggesting that HIF-1α transcriptional activity may be increased in primary uveal melanomas which metastasize [21]. LOX has also been linked to HIF-driven metastasis in multiple different tumor types and it is believed to promote metastasis by remodelling the extracellular matrix [22]–[24]. Recently, the increased expression of HIF-1α has been shown to be significantly associated with proliferative (MIB-1) and vascular (CD31 and VEGF-A) markers in uveal melanoma primary tumors, even though no significant correlation was observed between HIF-1α expression and patient survival [25].

Collectively, these studies implicate signaling pathways associated with low oxygen tension (hypoxia) in the spread of uveal melanoma. We examined the effects of oxygen level and HIF-1α expression on the growth and invasion of uveal melanoma cells in order to determine how the hypoxic response might modulate the biology of these tumors.

## Materials and Methods

### Cell lines, plasmids and reagents

Human cell lines derived from primary uveal melanoma, OCM1, Mel285, Mel290, 92.1 [26]–[29], or from a subcutaneous metastasis, OMM1 [30], were kindly provided by Dr. J. Niederkorn (UT Southwestern Medical Center, Dallas, TX), and cultured in RPMI 1640 medium as previously described [31], [32]. Lentiviral particles containing PLKO.1 transfer vector with short hairpin RNA (shRNA) targeting HIF-1α or CBF1 mRNA (target sequences are shown in Table S1) were purchased from Thermo Fisher Scientific (Waltham, MA) and prepared using HEK293T cells as previously described [31]. Retroviruses were generated using a pBABE vector carrying an oxygen stable mutant of HIF-1α HA-tagged (HIF-1α^Pro402Ala/Pro564Ala^), resistant to prolyl-hydroxylation and stably expressed in normoxia (Addgene, Cambridge, MA). pBABE vector alone was used as control. Puromycin (5 µg/mL) was used as selecting agent for both the gain- and loss-of-function experiments. Gamma-secretase inhibitor (GSI) MRK003 was provided by Merck & Co., Inc. [33]. Digoxin and rapamycin were purchased from Sigma-Aldrich (St. Louis, MO) and dissolved in DMSO. Erk1-2 inhibitor SCH772984 was provided by Merck & Co., Inc. and dissolved in DMSO [34]. For the hypoxia experiments, cell cultures were incubated for 24 hours in 1% pO_2_ atmosphere using an Oxygen Controller Glove Box (Coy Laboratory Products Inc., Grass Lake, MI), equilibrated with a mixture containing 1% oxygen, 5% carbon dioxide, and 94% nitrogen at 37°C.

### RNA extraction and quantitative real-time PCR

RNA from cell lines exposed to normoxia or hypoxia for 24 hours was isolated using the RNeasy Mini Kit (Qiagen, Germantown, MD) with on-column DNA digestion. Quantitative real-time PCR (qPCR) was carried out as previously described [31]. All reactions were performed in triplicate and each experiment was repeated three times, using an iQ5 Multicolor real-time PCR detection system (Bio-Rad, Hercules, CA), with SYBR Green (Applied Biosystems, Foster City, CA) as fluorescent dye, and normalized to β-Actin mRNA levels. Primer sequences for VEGF, LOX, and Notch pathway components were previously described [31], [35].

### Western blotting

Cellular cultures were exposed to normoxia or hypoxia for 24 hours, washed with cold PBS and lysed in TNE buffer (50 mM Tris-HCl, pH 7.4; 150 mM NaCl; 5 mM EDTA; 1% SDS) supplemented with protease inhibitor diluted 1∶100 (Sigma-Aldrich) and 1 mM sodium orthovanadate (New England BioLabs, Ipswich, MA). Protein lysates were sonicated on ice for 20 seconds, and their protein concentration measured using a bicinchoninic acid (BCA) assay kit (BCA Protein Assay Kit; Thermo Fisher Scientific/Pierce Protein Biology Products, Rockford, IL). Equivalent amounts of proteins were analysed by electrophoretic separation using SDS–polyacrylamide gels (Invitrogen, Carlsbad, CA). Proteins were transferred onto nitrocellulose membrane (Invitrogen) and exposed for 1 h to a blocking solution (5% dried milk in PBS). Filters were incubated overnight with the following antibodies specific for: HIF-1α (in mouse, 1∶800, BD Biosciences, #610959, San Jose, CA), HIF-2α (in rabbit, 1∶750, Novus Biologicals, #NB100–122, Littleton, CO), Hes1 (in rabbit, 1∶700, Aviva Systems Biology, #ARP32372, San Diego, CA), phospho-Ser^235/236^ S6 ribosomal protein (in rabbit, 1∶1000, Cell Signaling Technology, #4858, Danvers, MA), S6 ribosomal protein (in mouse, 1∶1000, Cell Signaling Technology, #2317), anti-cleaved Notch1 (in rabbit, 1∶1000, Cell Signaling Technology, #4147), phospho-Erk1-2^Thr202/Tyr204^, phospho-Akt^Ser473^, Erk1-2, Akt (in rabbit, 1∶1000, Cell Signaling Technology, #4376, #4060, #9102, #4691), anti-RBPSUH (CBF1) protein (in rabbit, 1∶800, Cell Signaling Technology, #5313), phospho-IkB-α^Ser32^ (in rabbit, 1∶1000, Cell Signaling Technology, #2859), β-Actin (in mouse, 1∶500, Sigma-Aldrich, #sc-47778, St. Louis, MO), or GAPDH (in mouse, 1∶5,000, RDI, #TRK5G4-6C5, Flanders, NJ). Peroxidase-coupled secondary antibodies raised in mouse or in rabbit (KPL, Gaithersburg, MD) were used to visualize protein bands. Enhanced chemiluminescence (ECL) was used as detection system (PerkinElmer, Waltham, MA).

### Cellular biology assays

#### Cell growth assay

Cell growth was determined using 3-(4,5-dimethylthiazol-2-yl)-5-(3-carboxymethoxy phenyl)-2-(4-sulfophenyl)-2H-tetrazolium (MTS) colorimetric assay (Promega, Madison, WI), as previously described [36]. Briefly, 5×10^3^ cells were seeded in each well in a 96-well plate and resuspended in 200 µL of 5% FBS medium per each well. Cell growth was measured every two days by adding 20 µL of MTS reagent/well and measuring the absorbance at λ = 490 nm in a microplate reader (BioTek, Winooski, VT), after 1 hour incubation at 37°C. The growth rate has been determined as follows: (Abs t – Abs t_0_)/n. days. Each experimental condition has been repeated in triplicate and data are presented as mean±standard deviation (SD).

#### Transwell invasion assay

Cellular invasion was analysed using 6.5-mm-diameter cell culture inserts (8-µm pore size; Becton Dickinson, Franklin Lakes, NJ) precoated for 1 hour with Matrigel (Becton Dickinson), diluted 1∶100 in 10% FBS medium, in 24-well plates. 1.8×10^5^ cells resuspended in serum free medium were plated in each filter insert, whereas 800 µL of 10% FBS medium were added to each well to create a chemoattractant gradient. After 24 hours cells that did not migrate were removed with a cotton swab from the upper surface of the filter, while those that moved to the lower surface of the filter were fixed with ethanol, stained with hematoxylin, destained, and photographed. Data represent the mean±SD of the number of cells counted in six random high-power fields (HPFs) in each of three independent experiments.

### Analysis of Primary Tumors

The study was approved by Johns Hopkins Medicine Institutional Review Board (IRB). Written informed consent was obtained from donors to use excess tumor tissues not required for diagnosis from primary uveal melanomas removed by enucleation at the Wilmer Eye Institute (Baltimore, MD). Eyes enucleated for uveal melanoma were grossly sectioned in the operating room. For each eye, a section containing a portion of the tumor was incubated in graded sucrose, snap frozen, and stored at −80°C.

### Immunohistochemistry

Streptavidin alkaline phosphatase (APase) immunohistochemistry was performed on cryopreserved tissue sections using a nitroblue tetrazolium (NBT) development system as previously described [37]. Rabbit anti-human HIF-1α (1∶7000, Abcam, Cambridge, MA) and mouse anti-human CD34 (Covance, Gaithersburg, MD), were used to detect HIF-1α protein and vascular endothelial cells, respectively.

### Statistical analysis

Experiments were performed in triplicate and data are presented as the mean±SD. Levels of significance were determined by 2-tailed Student’s t-test, with P values lower than 0.05 considered statistically significant. Statistical evaluations were carried out using GraphPad Prism4 software.

## Results

### HIF-1α is stabilized by both hypoxia and mTOR activity in uveal melanoma

**Figure 1:**
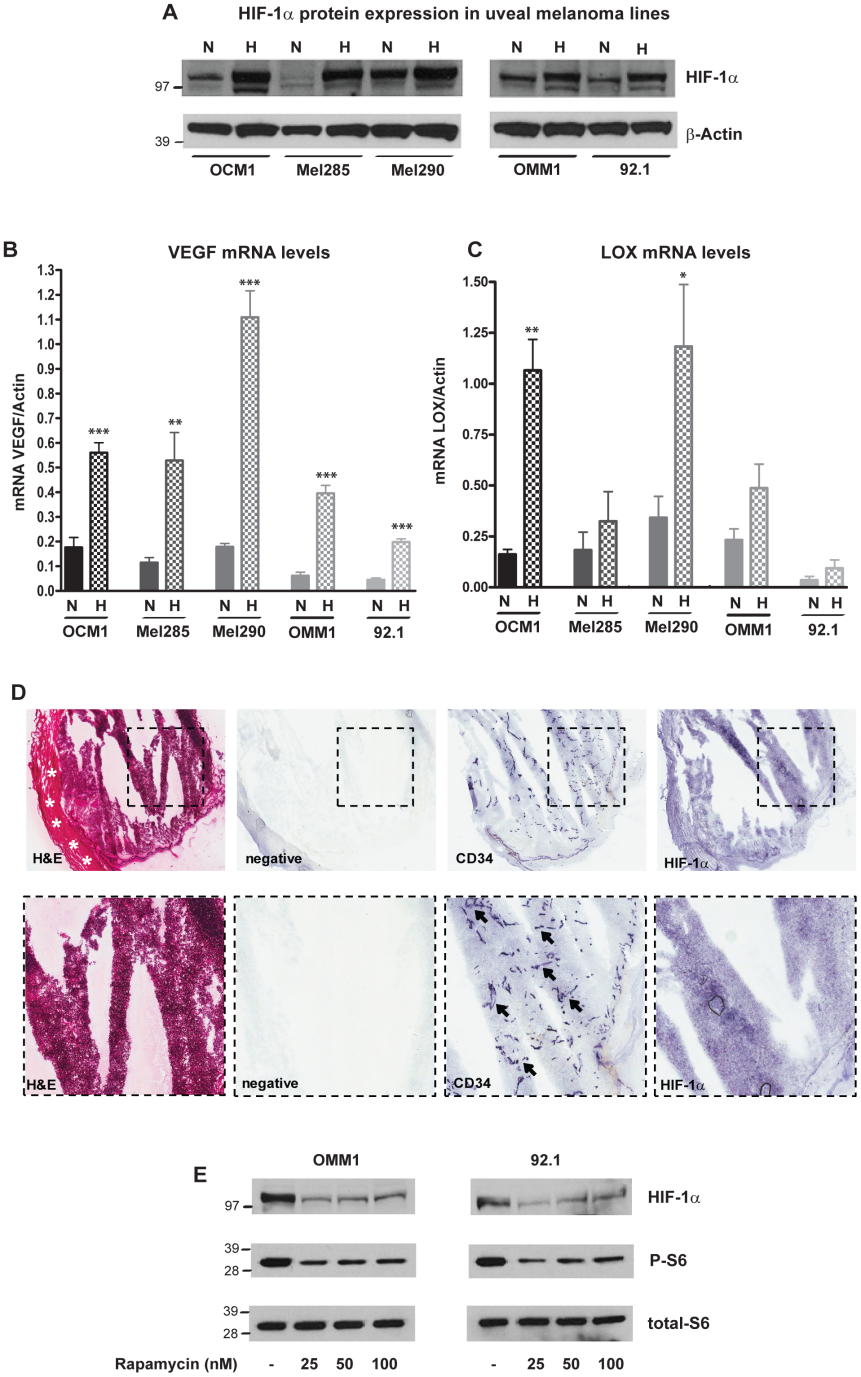
HIF-1α pathway is induced by hypoxia and mTOR activity in uveal melanoma. **A,** HIF-1α protein levels were determined by Western blot in OCM1, Mel285, Mel290, OMM1 and 92.1 lines grown in normoxia (N) or in hypoxia (H) for 24 hours; β-Actin was used as loading control. **B, C,** VEGF (B) and LOX (C) mRNA levels were analyzed by qPCR in five uveal melanoma lines after exposure for 24 hours to normoxia (N) or hypoxia (H), (*p = 0.02; **p = 0.005; ***p<0.0001). **D,** Expression of HIF-1α and CD34 was examined in snap frozen primary uveal melanoma specimens by immunohistochemistry. Scleral tissue is present on the left (asterisks) and tumor showing irregular cracking artefact due to freezing on the right. CD34 stains (arrows) highlight a dense capillary network, with diffuse HIF-1α staining in tumor tissue. **E,** HIF-1α, phospho-Ser^235/236^ S6 and total S6 ribosomal protein levels were determined by Western blot in OMM1 and 92.1 lines after 24 hours of treatment with rapamycin at 25, 50, 100 nM or DMSO; total ribosomal S6 protein was used for loading control.

To explore the role of HIF-1α in uveal melanoma, we first determined the baseline expression of HIF proteins and the mRNA levels of their downstream targets VEGF and LOX in uveal melanoma lines grown for 24 hours in normoxia (21% pO_2_) or hypoxia (1% pO_2_). HIF-1α protein was relatively abundant in the OCM1, OMM1, Mel290 and 92.1 uveal melanoma lines grown in normoxia, and was further induced by up to 5 fold in hypoxia (Figure 1A). In contrast, Western blot examination of HIF-2α protein in uveal melanoma lines revealed minimal expression in both low and normal oxygen tension (Figure S1). The mRNA levels of *VEGF* and *LOX* were also induced by 2 to 4 fold in hypoxia (Figure 1B, C). Thus HIF-1α protein expression is relatively elevated in many uveal melanoma lines under normoxic conditions, but its protein levels and transciptional activity are further induced when oxygen levels are lowered.

We also examined HIF-1α expression in five primary uveal melanoma specimens, which showed some irregular cracking due to artefacts related to freezing and sectioning. Using immunohistochemistry, we identified diffuse HIF-1α protein in all five cases (Figure 1D). We also stained sections for the vascular endothelial marker CD34, which highlighted a dense network of capillary vessels. Interestingly, several tumors contained well-vascularized regions in which we noted that HIF-1α protein was expressed around vessels, suggesting that it might also be present in normoxic regions of primary tumors.

It has been previously shown that in some neoplasms mTOR is an upstream activator of HIF-1α protein, enhancing its gene transcription [38]–[41]. We therefore investigated whether this might account for the robust HIF-1α levels noted in normoxic uveal melanoma cells. The mTOR inhibitor rapamycin was able to reduce both S6 phosphorylation and HIF-1α protein levels in normoxic tumor cells, suggesting that in uveal melanoma an active mTOR cascade may promote a “hypoxic” transcriptional response even in the presence of oxygen (Figure 1E).

### Hypoxia and HIF-1α promote uveal melanoma invasion

**Figure 2:**
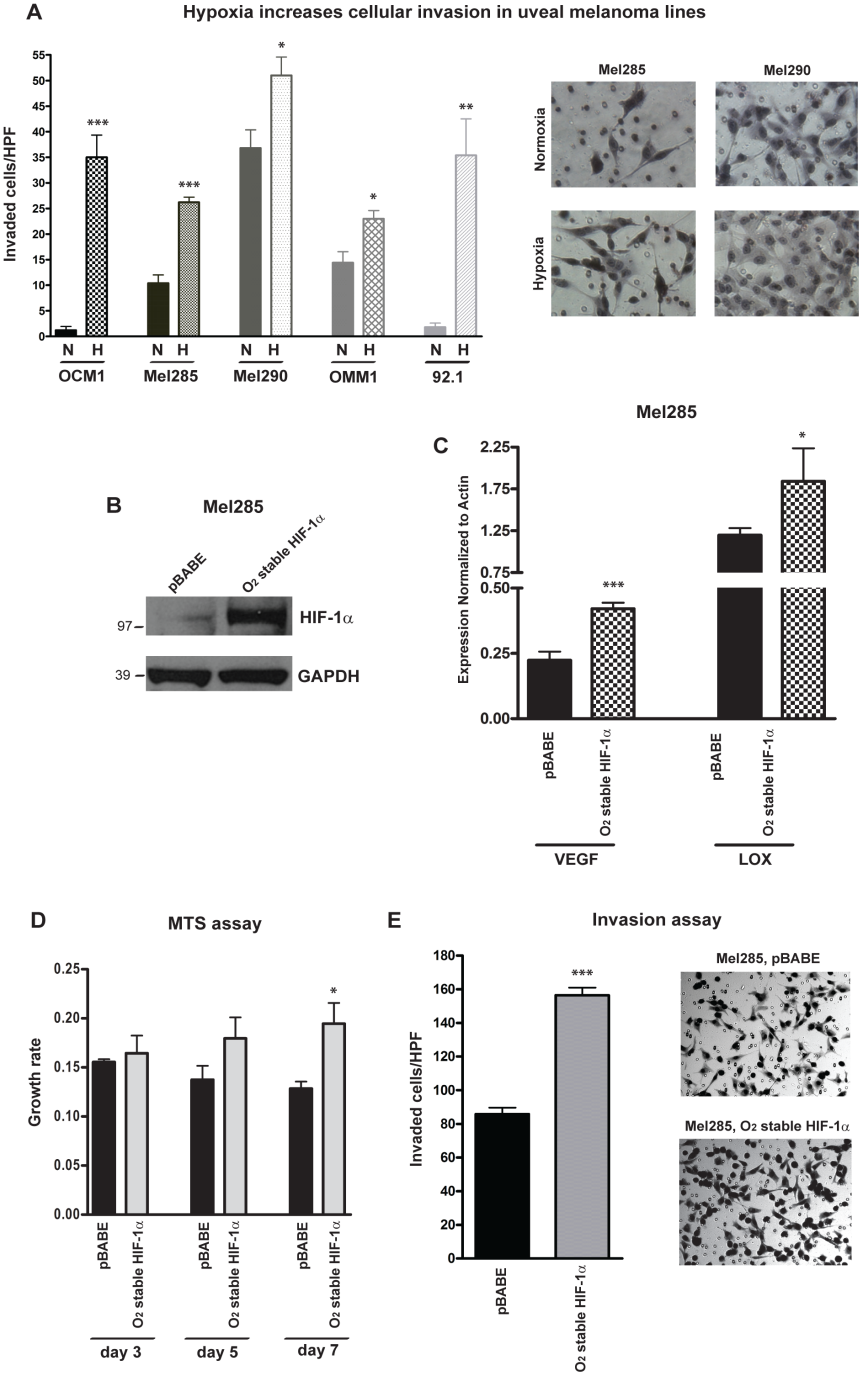
Upregulation of HIF-1α increases cellular invasion in uveal melanoma lines. **A,** Transwell invasion assay was performed in five uveal melanoma lines exposed to normoxia (N) or hypoxia (H) for 24 hours. In the microphotographs of the right panels the invading Mel285 or Mel290 cells are visualized on the lower surface of the Matrigel-coated filter after 24 hours of incubation in normal (21%) or low (1%) oxygen tension (*p = 0.02; **p = 0.002; ***p<0.0001). **B,** HIF-1α protein levels were determined by Western blot in Mel285 cells expressing an oxygen stable mutant of HIF-1α (HIF-1α^Pro402Ala/Pro564Ala^) or pBABE control vector. GAPDH was used as loading control. **C,** VEGF and LOX mRNA levels were analyzed by qPCR in Mel285 cells expressing HIF-1α oxygen stable mutant or pBABE vector (*p = 0.01; ***p<0.0001). **D,** MTS assay shows induction of cell growth in Mel285 cells infected with HIF-1α oxygen stable mutant compared to pBABE vector (*p = 0.04). **E,** Transwell invasion assay reveals increase of the invading capacity of Mel285 cells expressing HIF-1α oxygen stable mutant compared to control vector (***p<0.0001).

We next examined the effects of hypoxia on uveal melanoma invasion. When cultured in 1% oxygen, invasion through a membrane into Matrigel was significantly induced by 2 to 5 fold in all of the 5 cell lines tested (Figure 2A), suggesting that hypoxic factors may promote tumor spread. Mel285 cells have the lowest baseline level of HIF-1α compared to the other uveal melanoma lines (Figure 1A), therefore we used this line for additional gain-of-function studies. HIF-1α activity was induced in these cells by retroviral infection of an oxygen stable mutant of HIF-1α HA-tagged (HIF-1α^Pro402Ala/Pro564Ala^), which is resistant to VHL-mediated degradation and its expression is not reduced in normoxia. pBABE vector was used as control. We confirmed by Western blot the increase of HIF-1α protein in normoxia in Mel285 cells expressing the oxygen stable mutant of HIF-1α (Figure 2B), and we also observed by qPCR induction of *VEGF* and *LOX* mRNA expression in these cells as compared to the pBABE-infected cells (Figure 2C). Constitutive expression of HIF-1α in normoxia increased cell growth by approximately 35% (p = 0.04) as assessed by MTS assay (Figure 2D), and doubled the invasion rate through Matrigel (Figure 2E).

### HIF-1α suppression inhibits growth in hypoxia and invasion in both normoxia and hypoxia in uveal melanoma cells

**Figure 3:**
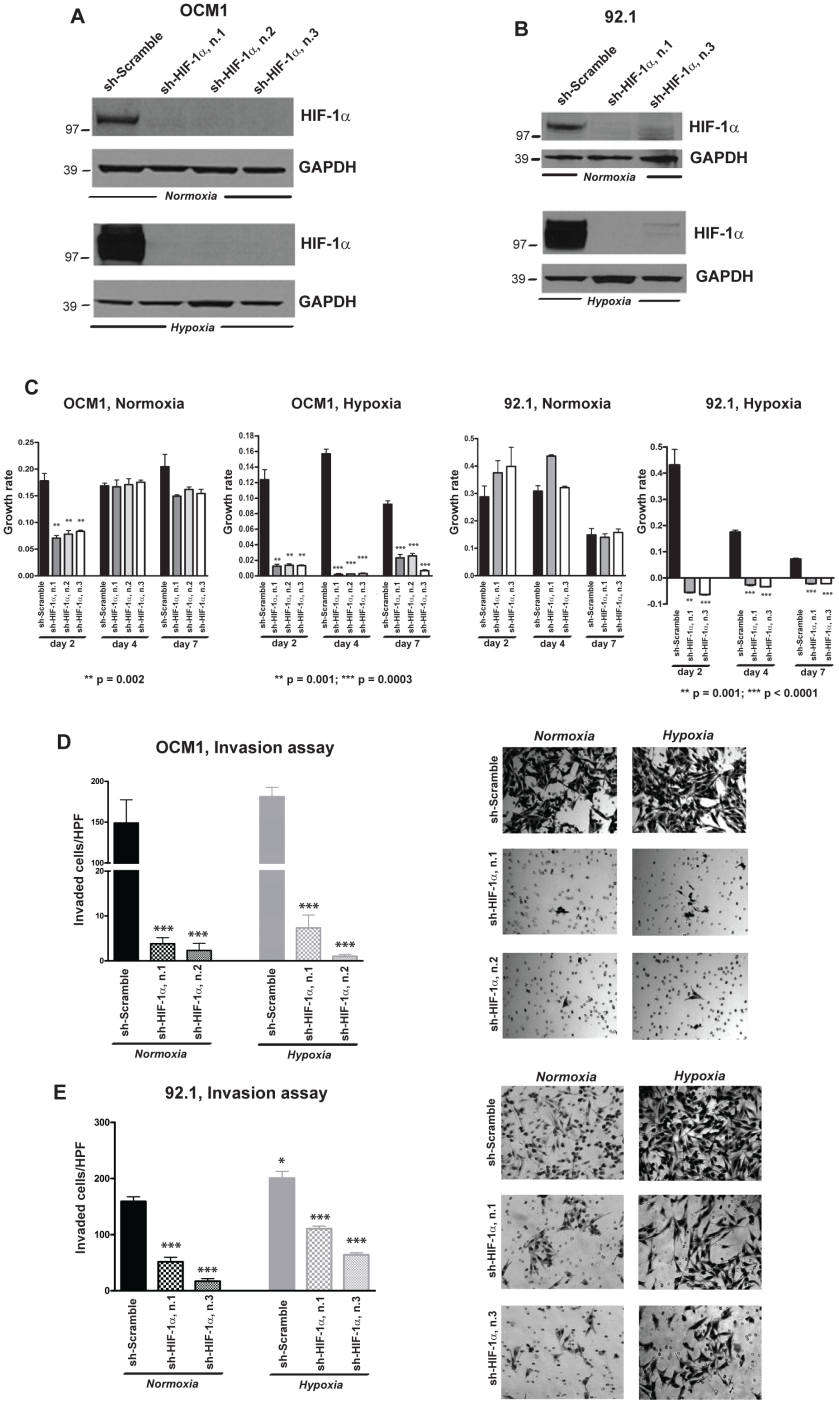
Downregulation of HIF-1α reduces cellular invasion in uveal melanoma lines. **A, B,** HIF-1α protein levels were determined by Western blot in OCM1 (A) and 92.1 cells (B) infected with HIF-1α or scramble shRNAs and exposed to normoxia or hypoxia for 24 hours. **C,** MTS assays were performed for 2, 4, 7 days in normoxia or hypoxia using OCM1 and 92.1 cells infected with HIF-1α or scramble shRNAs. P values were determined vs scramble control shRNA. **D, E,** Transwell invasion assay was carried out in OCM1 and 92.1 cells infected with HIF-1α or scramble shRNAs and exposed for 24 hours to normoxia or hypoxia (*p = 0.02; ***p = 0.0003).

In order to prove the importance of HIF-1α protein in the regulation of growth and invasion in uveal melanoma cells, we also performed loss-of-function studies. Since we observed that HIF-1α protein was relatively abundant even in normoxia in OCM1, Mel290, OMM1 and 92.1 cells (Figure 1A), we analyzed the effects that the downregulation of this protein has on cellular growth and invasion in these four lines. Three separate shRNA constructs targeting HIF-1α reduced protein expression by more than 95% both in normoxia and in hypoxia (Figure 3A, B, S2A, B). MTS assay showed that suppression of HIF-1α significantly reduced the rate of cellular growth after 7 days in culture under hypoxic conditions by 70–90% (p = 0.0003) in OCM1, by 130% (p<0.0001) in 92.1, by at least 50% (p = 0.016) in Mel290 and by 95% (p = 0.0009) in OMM1 cells. However, no significant reduction in cell growth was observed in normoxia in any of the lines analyzed, indicating the requirement of HIF-1α to support growth particularly in hypoxia (Figures 3C, S2C).

Downregulation of HIF-1α also produced a profund effect on the ability of the cells to migrate through Matrigel. Transwell invasion assays carried out in OCM1, Mel290, OMM1 and 92.1 cells transduced with HIF-1α or scramble shRNAs showed that the suppression of HIF-1α expression greatly impaired cellular invasion both in normoxia and hypoxia (p = 0.0006), suggesting that HIF-1α plays an important role in driving invasion under both oxygen conditions (Figure 3D, E, S2D, E).

### Digoxin inhibits cellular invasion in uveal melanoma cells

**Figure 4:**
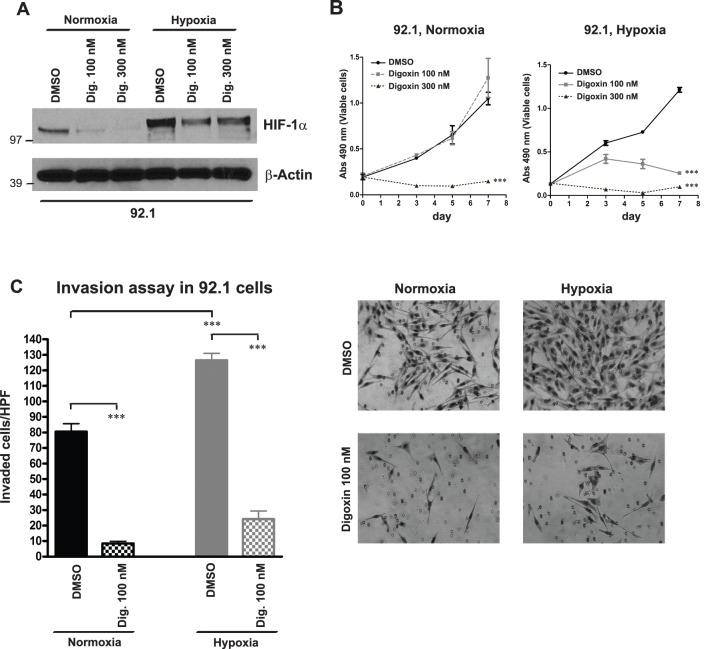
Digoxin inhibits cellular invasion in uveal melanoma cells. **A,** HIF-1α protein levels were determined by Western blot in 92.1 cells exposed to DMSO or digoxin at 100, 300 nM for 24 hours in normoxia or hypoxia. β-Actin was used as loading control. **B,** MTS growth assays were carried out in 92.1 cells treated with DMSO or digoxin at 100, 300 nM and exposed to normoxia or hypoxia for 3, 5, 7 days (***p<0.0001). **C,** Transwell invasion assay was performed in 92.1 cells treated with DMSO or digoxin at 100 nM and exposed to normoxia or hypoxia for 24 hours (***p<0.0001). The microphotographs in the right panel show the invading cells on the lower surface of the Matrigel-coated filter after 24 hours of incubation in normal (21%) or low (1%) oxygen tension.

Since digoxin, a cardiac glycoside used in the treatment of atrial fibrillation and heart failure, is also known to inhibit HIF-1α protein synthesis [41], we used this compound to pharmacologically suppress HIF-1α and analyze the effects on growth and invasion in uveal melanoma lines. While we observed a remarkable dose-dependent reduction of HIF-1α protein levels in 92.1 cells treated with digoxin at 100 and 300 nM for 24 hours in normoxia, we found that this decrease was attenuated in hypoxia (Figure 4A), perhaps due to the increase in the stability of the protein in the presence of low oxygen tension. Long-term treatment with digoxin for 3, 5, 7 days reduced in a dose-dependent manner cell growth in hypoxia, as found by MTS assay (p<0.0001), while in normoxia only the highest dose inhibited growth (Figure 4B). Interestingly, treatment with digoxin at 100 nM for 24 hours reduced by more than 4 fold the ability of the cells to invade Matrigel (Figure 4C), suggesting a more stringent role for HIF-1α in promoting invasion both in normoxia and in hypoxia.

### Hypoxia exposure activates the Notch pathway

**Figure 5:**
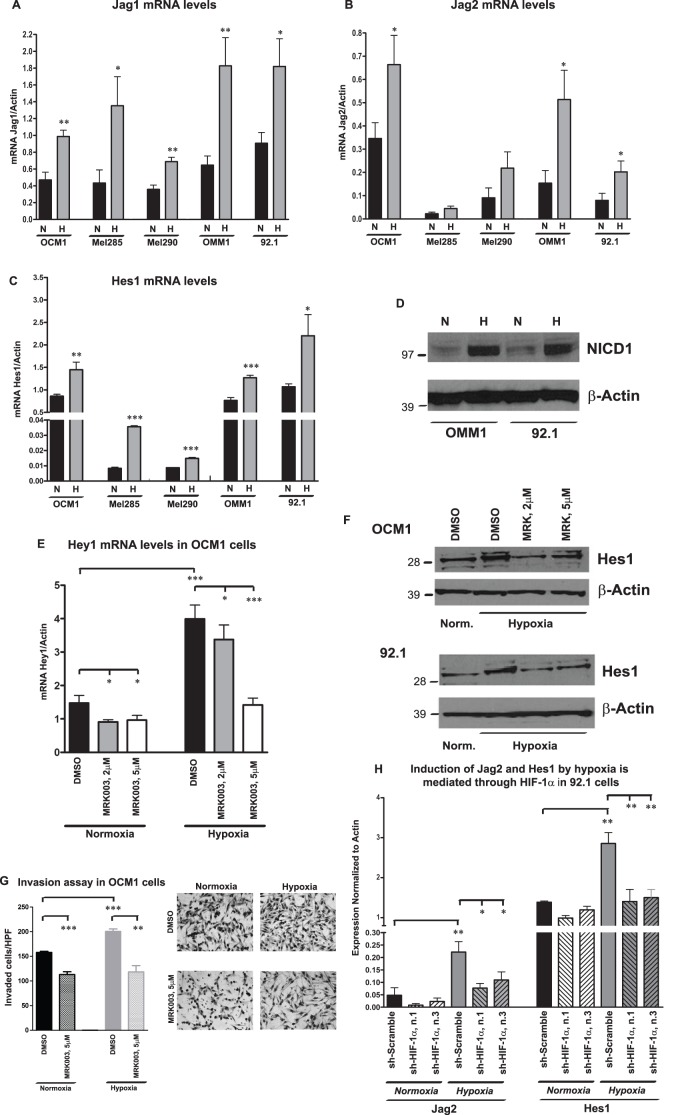
Hypoxia activates Notch pathway. **A–D,** Jag1 (A), Jag2 (B), Hes1 (C) mRNA levels were analyzed by qPCR in five uveal melanoma lines exposed to normoxia (N) or hypoxia (H) for 24 hours (*p = 0.01; **p = 0.001; ***p<0.0001). **D,** Notch1 intracellular domain (NICD1) was determined by Western blot in OMM1 and 92.1 cells exposed to normoxia (N) or hypoxia (H) for 24 hours. **E,** Hey1 mRNA levels were analyzed by qPCR in OCM1 cells pre-treated with MRK003 or DMSO for 24 hours and then exposed to hypoxia or normoxia for 24 hours (*p = 0.01; ***p<0.0001). **F,** Hes1 protein expression was analyzed by Western blot in OCM1 and 92.1 cells pre-treated with MRK003 or DMSO for 24 hours and then exposed to hypoxia or normoxia for 24 hours. **G,** Invasion assay was performed in OCM1 cells exposed to 5 µM of MRK003 or DMSO for 24 hours in normoxia or hypoxia (**p = 0.001; ***p = 0.0002). **H,** Jag2 and Hes1 mRNA levels were determined by qPCR in 92.1 cells infected with HIF-1α or scramble shRNAs and exposed to normoxia or hypoxia for 24 hours (*p = 0.02; ***p<0.0001).

In order to determine downstream targets of HIF-1α responsible for these profound effects on cellular invasion, we examined the expression of Notch pathway members, which we have previously linked to the spread of uveal melanoma cells [31], [32]. Interestingly, hypoxia induced at least a 2 fold increase in the mRNA expression of the Notch ligands *Jag1* and *Jag2* as determined by qPCR (Figure 5A, B). We also observed a significant increase in the expression of *Hes1* and *Hey1* target genes after 24 hours exposure to hypoxia (Figure 5C, S3). As further confirmation that Notch signaling is induced by low oxygen tension, we found a striking increase in the protein levels of the intracellular domain of Notch1 receptor (NICD1) in hypoxia as compared to normoxia (Figure 5D).

To determine if the induction of Notch targets in reduced oxygen tension was due to canonical pathway activity, we exposed OCM1 and 92.1 cells to the γ-secretase inhibitor MRK003 at 2 and 5 µM for 48 hours, with hypoxia or control growth conditions over the last 24 hours of this treatment. The hypoxia-induced increase in *Hes1* and *Hey1* mRNA levels was almost completely blocked by the higher level of MRK003, supporting the hypothesis that canonical, γ-secretase mediated Notch signaling is being activated under hypoxic conditions (Figure 5E, S4A for OCM1, Figure S4B for 92.1). The increase of Hes1 by hypoxia was also confirmed at the protein level, and this induction was reduced by MRK003 (Figure 5F). Pharmacological blockade of Notch signaling prevented the induction of cellular invasion due to hypoxia, as determined by transwell invasion assay performed in OCM1 and 92.1 cells treated with MRK003 at 5 µM, and exposed to hypoxia or normoxia for 24 hours (Figure 5G, S5).

To further investigate the mechanism of crosstalk between hypoxia and Notch signaling, we analyzed Notch pathway components in 92.1 cells where HIF-1α was genetically suppressed by two separate sh-HIF-1α constructs. We observed that such suppression correlated with a reduction in the mRNA levels of *Jag2* ligand in normoxia, and prevented Jag2 induction by hypoxia compared to scramble shRNA. Also the hypoxia-dependent induction of *Hes1* mRNA was suppressed by sh-HIF-1α constructs (Figure 5H). Thus HIF-1α appears to activate canonical Notch signaling, including the target Hes1, in hypoxia by inducing the expression of pathway ligands such as Jag2.

**Figure 6:**
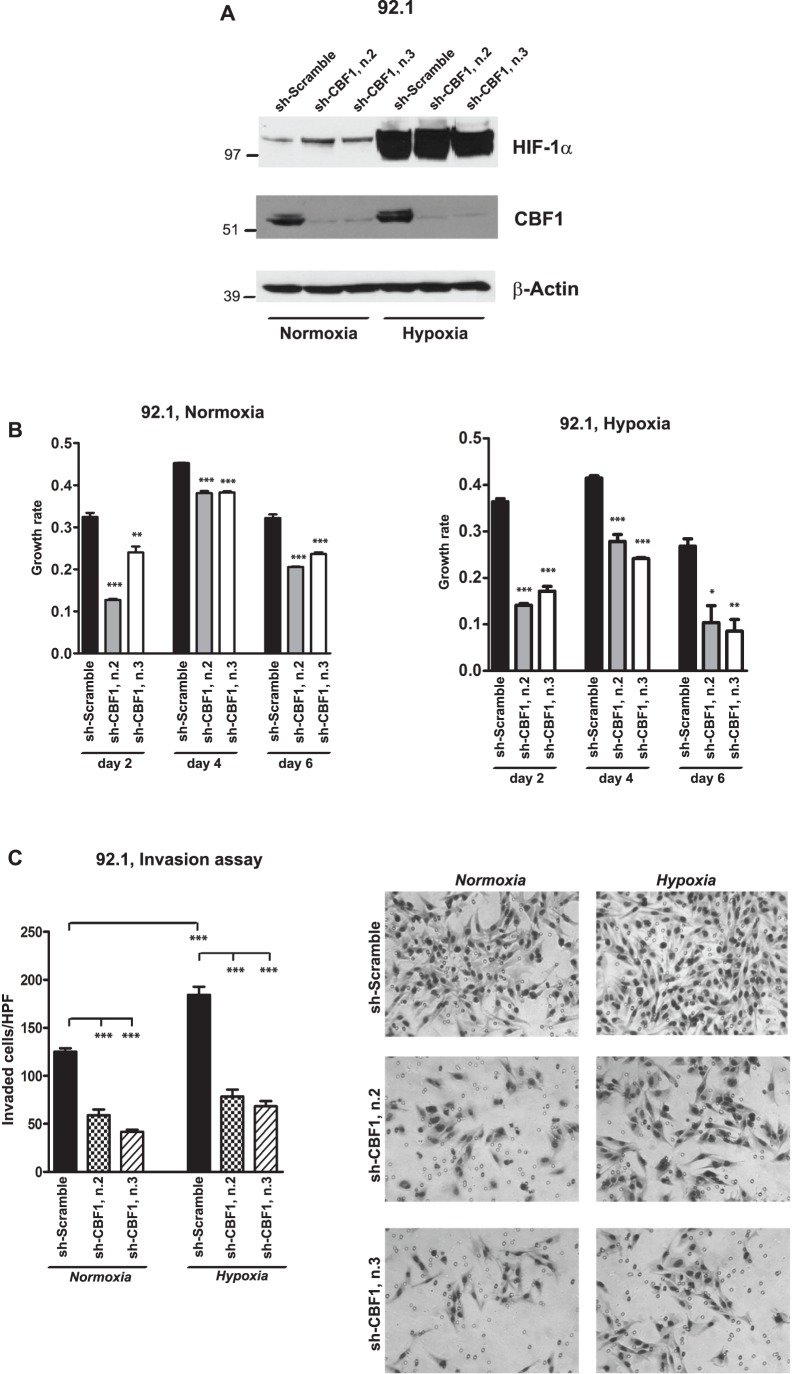
Genetic inhibition of Notch signaling reduces hypoxia-mediated cellular invasion. **A,** The protein levels of HIF-1α and CBF1 were determined by Western blot in 92.1 cells infected with CBF1 or scramble shRNAs and exposed to normoxia or hypoxia for 24 hours; β-Actin was used as loading control. **B,** Growth rate was determined by MTS assay in 92.1 cells infected with CBF1 or scramble shRNAs and exposed to normoxia or hypoxia for 2, 4, 6 days (*p = 0.01; **p = 0.001; ***p<0.0001). **C**, Transwell invasion assay was performed in 92.1 cells infected with CBF1 or scramble shRNAs and exposed for 24 hours to normoxia or hypoxia (***p<0.0001).

To additionally prove the requirement of Notch in promoting cellular invasion upon hypoxia exposure in uveal melanoma cells, we genetically inhibited Notch signaling in 92.1 cells, which we have previously shown to have high Notch activity [32], using shRNAs that specifically target CBF1, one of the main components of canonical Notch signaling. Two separate constructs potently inhibited CBF1 protein expression (Figure 6A). In sh-CBF1-infected cells we observed a significant reduction in cell growth and more than two-fold decrease in invasion as compared to scramble sh-RNA, both in normoxia and in hypoxia, indicating an essential role for Notch in promoting growth and invasion under both oxygen tension conditions (Figures 6B, C).

### Hypoxia exposure activates Akt and MAPK signaling

**Figure 7:**
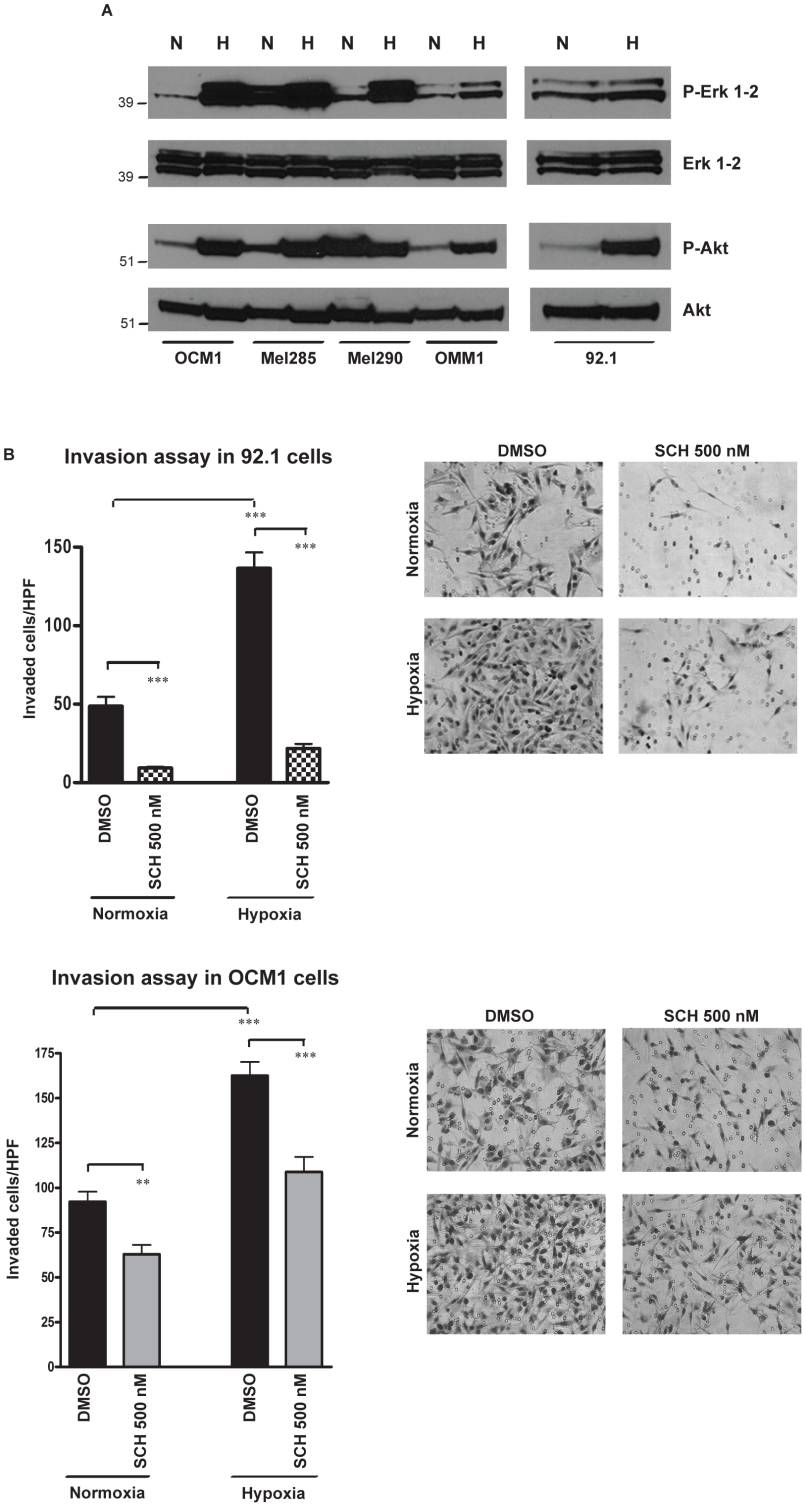
Hypoxia activates MAPK and Akt pathways. **A,** Western blot analysis reveals that exposure to hypoxia for 24 hours activates Erk1-2 and Akt proteins in all the uveal melanoma lines, as found using antibodies specific for phospho-Erk1-2^Thr202/Tyr204^ and phospho-Akt^Ser473^; total Erk1-2 and Akt were used as loading controls. **B**, Transwell invasion assay was performed in 92.1 and OCM1 cell lines treated with the Erk1-2 inhibitor SCH772984 at 500 nM, while exposed to normoxia or hypoxia for 24 hours. The microphotographs of the right panels show the invading cells on the lower surface of a Matrigel-coated filter after 24 hours of incubation in normal (21%) or low (1%) oxygen tension in the presence of the Erk1-2 inhibitor (***p<0.0001).

**Figure 8:**
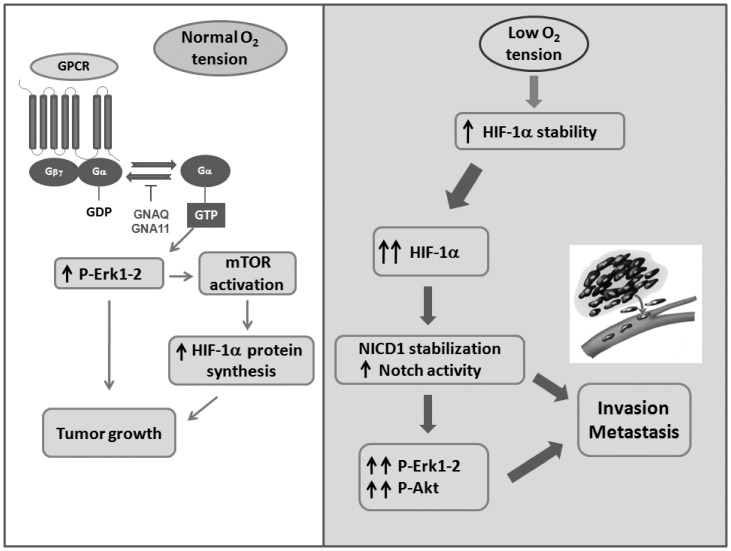
Working model. The working model shows that under normal oxygen tension GNAQ/GNA11 mutations, present in the G_α_ subunit of heterotrimeric G proteins (GPCR: G protein-coupled receptors) in the majority of primary uveal melanomas, are responsible for the activation of MAPK pathway under normoxic conditions. MAPK pathway in turn activates mTOR signaling, known to regulate HIF-1α protein translation and tumor growth. In hypoxic conditions, HIF-1α protein levels are further induced by the inhibition of its oxygen-dependent degradation. Such induction leads to Notch1 intracellular domain (NICD1) stabilization and Notch activation. The induction of P-Erk and P-Akt, mediated by a non-canonical Notch signaling, contributes in promoting invasion and metastasis.

Interestingly, we observed that in uveal melanoma cells exposure to hypoxia for 24 hours activates Akt and Erk1-2 proteins, as found by Western blot using antibodies specific for phospho-Erk1-2^Thr202/Tyr204^ and phospho-Akt^Ser473^ (Figure 7A). Such activation was in part mediated by Notch signaling, since it was partially inhibited by the γ-secretase inhibitor MRK003 (Figure S6A) and by sh-CBF1 (Figure S6B). We used the Erk inhibitor SCH772984 to suppress the phosphorylation of Erk1-2 in 92.1 and OCM1 cells (Figure S7). Interestingly we observed that the treatment of these cells with SCH772984 at 500 nM for 24 hours significantly reduced the number of cells invading a Matrigel-coated membrane either in normoxic and in hypoxic conditions (Figure 7B). Based on these data, we propose a model in which GNAQ/GNA11 mutations, detected in the Gα subunit of heterotrimeric G proteins in the majority of primary uveal melanomas, are responsible for the activation of MAPK pathway under normoxic conditions. Erk1-2 in turn promotes the activation of mTOR signaling, known to regulate HIF-1α protein translation and tumor growth. In hypoxic conditions, reached when the tumor mass outgrows the available blood supply, resulting in areas of low oxygen tension, a more profound increase of HIF-1α protein levels is induced by the inhibition of its oxygen-dependent degradation. Such induction leads to stabilization of Notch1 intracellular domain (NICD1) and Notch activation. The stimulation of Erk and Akt, mediated by a non-canonical Notch pathway, contributes in promoting invasion and metastasis (Figure 8).

## Discussion

Metastatic progression represents the major cause of death due to uveal melanoma. Even though 98% of patients do not show signs of metastatic dissemination at the time of diagnosis, about 50% of them will develop metastatic disease, which is thus far untreatable [42]. Gene expression analysis of primary uveal melanomas has revealed a number of pathways potentially responsible for the metastatic spread, including the hypoxic signaling mediated by hypoxia-inducible factor 1α (HIF-1α) and its targets [13], [21]. This is also supported by a previous study which shows that exposure to low oxygen tension increases migration, invasion and adhesion of Mum2B uveal melanoma cells, activating the expression of CXCR4, angiopoietin-related protein, and pyruvate dehydrogenase kinase 1 in a HIF-1-dependent manner [43]. Hypoxia and HIF-1α have also been linked to the tumorgenicity of cutaneous melanoma [44]–[46]. In addition it has been shown that VEGF levels are significantly increased in uveal melanoma patients with metastatic disease as compared to patients without metastases, therefore using anti-angiogenic therapies might be potentially beneficial for the treatment of primary uveal melanoma or metastatic disease [47]. We therefore sought to extend these promising initial findings with additional gain- and loss-of-function analyses in multiple uveal melanoma lines, analysis of downstream pathways activated by hypoxia, and investigation of pharmacological inhibitors potentially useful for clinical intervention.

Interestingly, we observed that HIF-1α protein is stabilized even in normoxia in most of the uveal melanoma lines examined, broadening the type of microenvironment where targeting HIF would be effective. HIF-1α stabilization in normoxia can be due to sustained activation of the mTOR pathway, which was previously shown to be responsible for regulating the translation of HIF-1α mRNA in other tumor types [38]–[41]. Treatment with the mTOR inhibitor rapamycin reduced HIF-1α protein levels in normal oxygen tension in OMM1 and 92.1 cells, supporting a similar paradigm in uveal melanoma cells. Our data also indicate that baseline expression of HIF-1α under normoxic conditions may play an important role in promoting cellular invasion, since pharmacological and genetic downregulation of HIF-1α reduced the invasion rate in both normoxia and hypoxia. Thus the physiological role of HIF-1α in normoxia is to promote invasion and this function is maintained and even enhanced in hypoxia.

We performed HIF-1α loss-of-function studies in multiple uveal melanoma cell lines, since recent mutational profile studies have shown that BRAF^V600E^ mutation, which is pretty rare in primary uveal melanomas, but more frequent in cutaneous melanomas, has been detected in a subset of uveal melanoma cell lines, including OCM1. This finding implies that the lines harboring such mutation and lacking of the GNAQ or GNA11 mutations, which are present in about 80% of the primary uveal melanomas, should be considered non-typical uveal melanoma cell lines [48].

To determine downstream pathways activated by hypoxia and responsible for the strong increase of cellular invasion we examined Notch signaling, which we have previously demonstrated to be active in many primary uveal melanoma and responsible for the induction of cellular invasion and clonogenic growth in uveal melanoma lines [31], [32]. Notch1 signaling has also been found to be responsible for the progression of primary cutaneous melanoma [49]. We found that the Jag1 and Jag2 ligands, the activated intracellular domain of the receptor, and the Hes1 and Hey1 target genes were all induced by hypoxia exposure.

Pharmacological suppression of the Notch pathway using the γ-secretase inhibitor MRK003 abrogated the hypoxia-dependent induction of Hes1 and Hey1, and prevented the increase of cellular invasion due to hypoxia, suggesting that canonical Notch signaling is activated by low oxygen levels. Genetic suppression of HIF-1α by shRNA prevented both Jag2 and Hes1 induction by hypoxia, further corroborating involvement of the Notch pathway as a downstream target of hypoxia-induced signaling.

At least two mechanisms might drive this Notch pathway induction. Previous studies have shown that HIF-1α can directly stabilize the activated Notch1 intracellular domain (NICD1), thereby increasing signaling in hypoxia [50]. However, we also see increased Jag1 and Jag2 in hypoxia, suggesting that HIF-1α may activate Notch signaling in part through ligand induction. Indeed, a recent study demonstrated HIF binding to a potential hypoxic response element (HRE) downstream of the Jag2 start codon, supporting a direct mechanism for the ligand induction we observed [51]. Our overall findings are consistent with the observation of elevated Notch signaling during hypoxia in many different tumor types [35], [50]–[52].

In cutaneous melanoma, it has been found that Notch1 promotes tumor progression by activating MAPK and Akt pathways [48], which have been linked to cellular invasion and metastatic spread in different tumor types [53], [54]. However, in uveal melanoma cells we observed that hypoxia exposure potently activates Erk1-2 and Akt pathways, partially through non-canonical Notch signaling. We also found that pharmacological inhibition of Erk1-2 signaling produced a more than two-fold reduction in the ability of 92.1 and OCM1 cells to invade a Matrigel-coated membrane both in normoxic and in hypoxic conditions, and suppressed the hypoxia-mediated induction of cellular invasion, suggesting that MAPK signaling plays a role in the increase of cellular invasion due to hypoxia exposure. In Mel285 cells, we detected very high levels of phospho-Erk1-2 in normoxia, which were further induced in hypoxia (Figure 7A). This high baseline activity of Erk1-2 in Mel285 cells might explain why this line has the second highest level of cellular invasion (Figure 2A), even though it shows little detectable HIF-1α level in normoxia (Figure 1A). A previous report shows that Src expression is associated with MAPK activation in some uveal melanoma lines, including Mel285, and this observation might explain the sustained induction of Erk1-2 that we observed at the steady state in this line [55].

Previous studies have shown that NF-kB activity contributes to HIF-1α accumulation in cutaneous melanoma [56]. However in uveal melanoma lines we did not see any induction in the phosphorylation of IkB-α^Ser32^, a marker of NF-kB activation, under hypoxic conditions (data not shown).

In summary, we demonstrate that HIF-1α is expressed in both normoxic and hypoxic uveal melanoma cell lines, as well as in primary tumor regions rich in blood vessels. Suppressing HIF-1α expression using either shRNA or pharmacological blockade has negative effects on tumor growth and invasion, suggesting that targeting the hypoxic response may be clinically beneficial. Downstream pathways that we found activated by hypoxia exposure include Notch, Erk1-2 and Akt, and we found that activation of Notch and MAPK was required for full induction of cellular invasion under hypoxic conditions. Since several uveal melanoma lines appear to have a baseline expression of HIF-1α even in normoxia, which is further induced by hypoxia, we propose that pharmacological compounds inhibiting HIF-1α pathway might be potentially useful for therapeutic intervention in preventing the metastatic spread of primary uveal melanoma even in a microenvironment characterized by normal oxygen tension.

## Supporting Information

Figure S1 **HIF-2α protein levels were determined by Western blot in uveal melanoma lines grown in normoxia (N) or in hypoxia (H) for 24 hours; β-Actin was used as loading control.**
(EPS)

Figure S2 **A, B,** HIF-1α protein levels were determined by Western blot in Mel290 (A) and OMM1 cells (B) infected with HIF-1α or scramble shRNAs and exposed to normoxia or hypoxia for 24 hours. **C,** MTS assays were performed for 2, 4, 7 days in normoxia or hypoxia using Mel290 and OMM1 cells infected with HIF-1α or scramble shRNAs. P values were determined vs scramble control shRNA. **D, E,** Transwell invasion assay was carried out in Mel290 and OMM1 cells infected with HIF-1α or scramble shRNAs and exposed for 24 hours to normoxia or hypoxia (**p = 0.006; ***p<0.0006).
(EPS)

Figure S3 Hey1 mRNA levels were analyzed by qPCR in five uveal melanoma lines exposed to normoxia (N) or hypoxia (H) for 24 hours (*p = 0.01; ***p<0.0002).
(EPS)

Figure S4 Hes1 mRNA levels were analyzed by qPCR in OCM1 cells (A), while Hes1 and Hey1 mRNA values were determined by qPCR in 92.1 cells (B) pre-treated with MRK003 or DMSO for 24 hours and then exposed to hypoxia or normoxia for 24 hours (*p = 0.01; **p = 0.001; ***p<0.0001).
(EPS)

Figure S5 Invasion assay was performed in 92.1 cells exposed to 5 µM of MRK003 or DMSO for 24 hours in normoxia or hypoxia (***p = 0.0001). The microphotographs of the right panel represent the invading cells visualized on the lower surface of the Matrigel-coated filter after 24 hour incubation.
(EPS)

Figure S6 **A,** Western blot was carried out using antibodies specific for phospho-Erk1-2^Thr202/Tyr204^, phospho-Akt^Ser473^, and β-Actin in OCM1 cells treated with the γ-secretase inhibitor MRK003 at 2 and 5 µM for 96 hours, with exposure to hypoxia or normoxia over the last 24 hours of this treatment. **B,** Protein levels of CBF1, phospho-Erk1-2^Thr202/Tyr204^, phospho-Akt^Ser473^, and β-Actin were determined by Western blot in 92.1 cells infected with CBF1 or scramble shRNAs and exposed to normoxia or hypoxia for 24 hours.
(EPS)

Figure S7 **Western blot was carried out using antibodies specific for HIF-1α, phospho-Erk1-2^Thr202/Tyr204^, and Erk1-2 in 92.1 and OCM1 cells exposed to normoxia or hypoxia for 24 hours and then treated with Erk1-2 inhibitor SCH772984 at 500 nM for 1 hour.**
(EPS)

Table S1 **Target sequences for the short hairpin RNA (shRNA) targeting HIF-1α or CBF1 mRNA.**
(EPS)
